# Techniques and graft materials for repairing peripheral nerve defects

**DOI:** 10.3389/fneur.2023.1307883

**Published:** 2024-01-22

**Authors:** Xiaodi Zou, Yanzhao Dong, Ahmad Alhaskawi, Haiying Zhou, Sohaib Hasan Abdullah Ezzi, Vishnu Goutham Kota, Mohamed Hasan Abdulla Hasan Abdulla, Sahar Ahmed Abdalbary, Hui Lu, Changxin Wang

**Affiliations:** ^1^Department of Orthopedics, The Second Affiliated Hospital of Zhejiang Chinese Medical University, Hangzhou, China; ^2^Department of Orthopedics, The First Affiliated Hospital, Zhejiang University, Hangzhou, China; ^3^Department of Orthopedics, Third Xiangya Hospital, Central South University, Changsha, China; ^4^Zhejiang University School of Medicine, Hangzhou, China; ^5^Department of Orthopedic Physical Therapy, Faculty of Physical Therapy, Nahda University in Beni Suef, Beni Suef, Egypt; ^6^Alibaba-Zhejiang University Joint Research Center of Future Digital Healthcare, Zhejiang University, Hangzhou, China

**Keywords:** peripheral nerve injuries, graft materials, peripheral nerve defects, nerve regeneration, nerve gap

## Abstract

Peripheral nerve defects refer to damage or destruction occurring in the peripheral nervous system, typically affecting the limbs and face. The current primary approaches to address peripheral nerve defects involve the utilization of autologous nerve transplants or the transplantation of artificial material. Nevertheless, these methods possess certain limitations, such as inadequate availability of donor nerve or unsatisfactory regenerative outcomes post-transplantation. Biomaterials have been extensively studied as an alternative approach to promote the repair of peripheral neve defects. These biomaterials include both natural and synthetic materials. Natural materials consist of collagen, chitosan, and silk, while synthetic materials consist of polyurethane, polylactic acid, and polycaprolactone. Recently, several new neural repair technologies have also been developed, such as nerve regeneration bridging technology, electrical stimulation technology, and stem cell therapy technology. Overall, biomaterials and new neural repair technologies provide new methods and opportunities for repairing peripheral nerve defects. However, these methods still require further research and development to enhance their effectiveness and feasibility.

## Introduction

Peripheral nerve injuries (PNIs) refer to damage to the peripheral nervous system, which includes all nerves outside of the brain and spinal cord (1). A variety of factors, such as trauma, compression, and disease, can lead to these injuries (2–5). The prevalence of PNIsis approximated to be within the range of 13 and 23 individuals per 100,000 annually in developed nations, causing either incomplete or complete deprivation of motor, sensory, and autonomic abilities in the affected regions of the anatomy (6, 7). The importance of the nerve damage relies on the extent and intensity of the sensory or motor impairment, the length of time the clinical symptoms persist, and the individual affected by the nerve injury (1).

The nerves are encased by the epineurium, perineurium, and endoneurium, each playing a crucial role. The epineurium acts as a protective shield for the nerve against external stressors. Situated beneath the epineurium, the perineurium consists of a thin layer of flat cells with tight junctions, serving to control diffusion around individual fascicles and exhibiting high tensile strength. The endoneurium, which is characterized by a relaxed collagen matrix, envelops individual nerve fibers (8).

**Figure 1:**
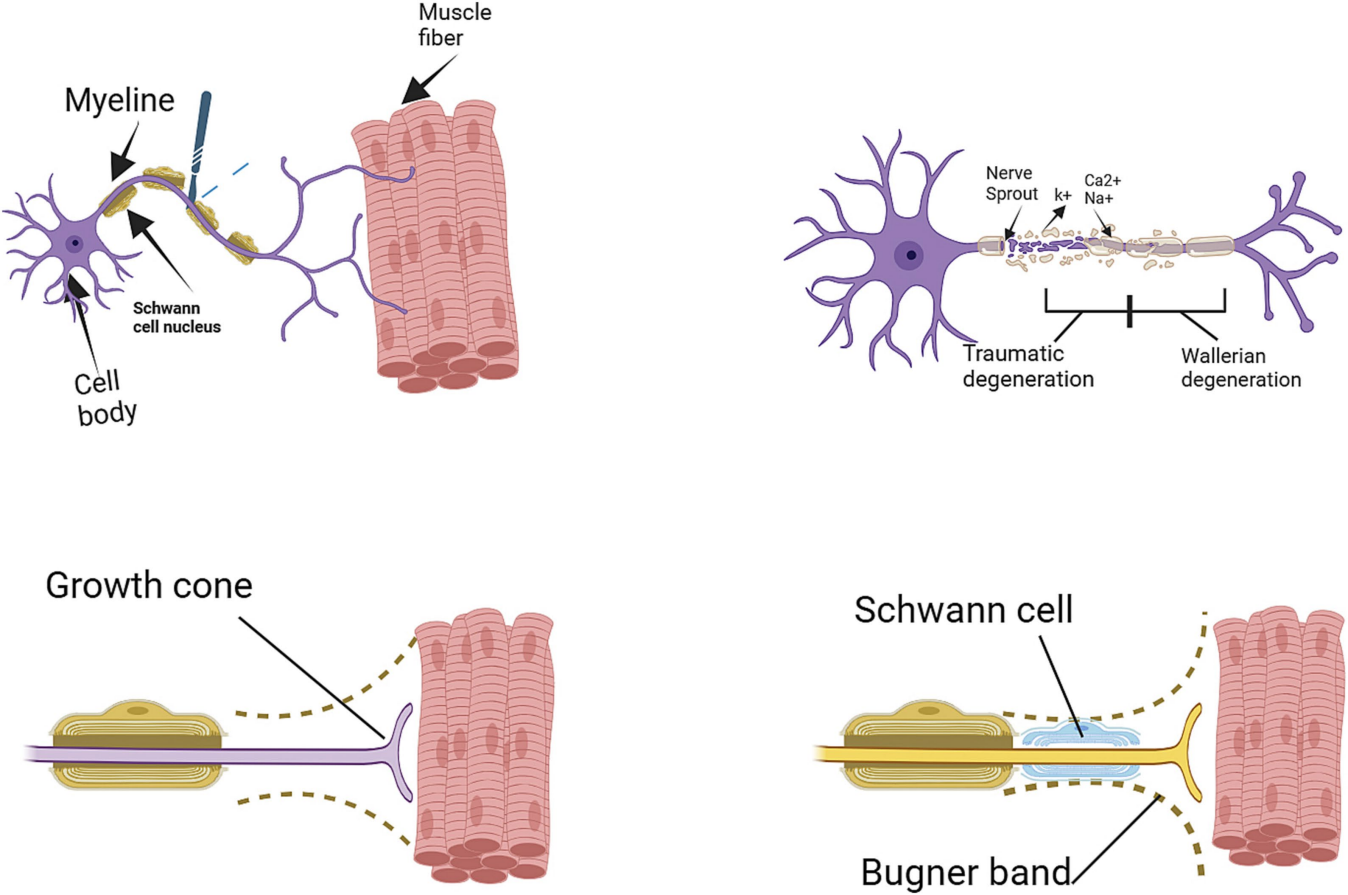
Degeneration and regeneration after peripheral nerve injury (11).

The process of nerve regeneration is intricate, encompassing multiple phases such as degeneration, sprouting, and reinnervation. In the wake of nerve injuries, axonal degeneration ensues, which is characterized by the disintegration of damaged axons and their myelin sheath. Schwann cells, located in the nerve’s distal segment, initiate the catabolism of myelin and phagocytosis of the debris. Within a day post-injury, the axonal sprouting commences from the injured nerve’s proximal stump. The growth cone of the sprouting axon progresses along the path of the unscathed basal lamina, a process influenced by neurotrophic and neurite-promoting factors. Assuming the endoneurial tube remains intact, the regenerating axon can follow a direct path to the end organ, migrating at an approximate rate of 1 mm per day. Neurotrophic factors are paramount in supporting peripheral nerve regeneration. Successful reinnervation activates both old and new motor end plates, facilitating muscle recovery. Given an appropriate route, peripheral axons have the capacity to regenerate and establish connections with their intended targets (9). However, in the absence of a suitable path, neuroma and scar tissue may form at the damaged nerve’s proximal end, obstructing nerve regeneration progress. The perineurium plays an essential role in maintaining axonal integrity; without it, axon fibers fail to proceed as expected. Axons are likely to deviate from their path once the perineurium sustains damage. Neuroinflammation is another pivotal factor in this process. When axons reach the extraperineurial space – an area already subjected to tissue damage – inflammation ensues. Substances secreted during this inflammatory response can contribute to the development of neuromas (5). Schwann cells and stem cells in the area affected by injury can preserve their survival through autocrine circuits, which inhibit apoptosis in dense environments, thereby enhancing the probability of axonal growth from the proximal area towards the distal stump (10). Regardless of this regenerative potential, peripheral nerve regeneration often results in suboptimal functional outcomes, largely due to the significant gap between severed injured peripheral nerves and their intended targets, which hampers reconnection (10, 11) (see Figure 1).

Neurotmesis corresponds to Sunderland’s classification of fifth-degree injury, which denotes the most extreme type of peripheral nerve damage characterized by a total interruption of the nerve (12). Surgery is always required to treat neurotmesis. A nerve defect, also known as a large nerve gap, cannot be directly repaired by suturing. The timely diagnosis of peripheral nerve injuries holds significant importance in their subsequent treatment. Conventional methods like MRI and ultrasound have been extensively utilized for diagnosing peripheral nerve injuries. Nevertheless, the intricate nature of MRI interpretation poses challenges, thereby restricting its practical implementation in clinical settings. In light of this, a recent study has introduced an end-to-end learning framework that leverages automatic image segmentation technology to streamline the process of MRI interpretation (13).

**Figure 2:**
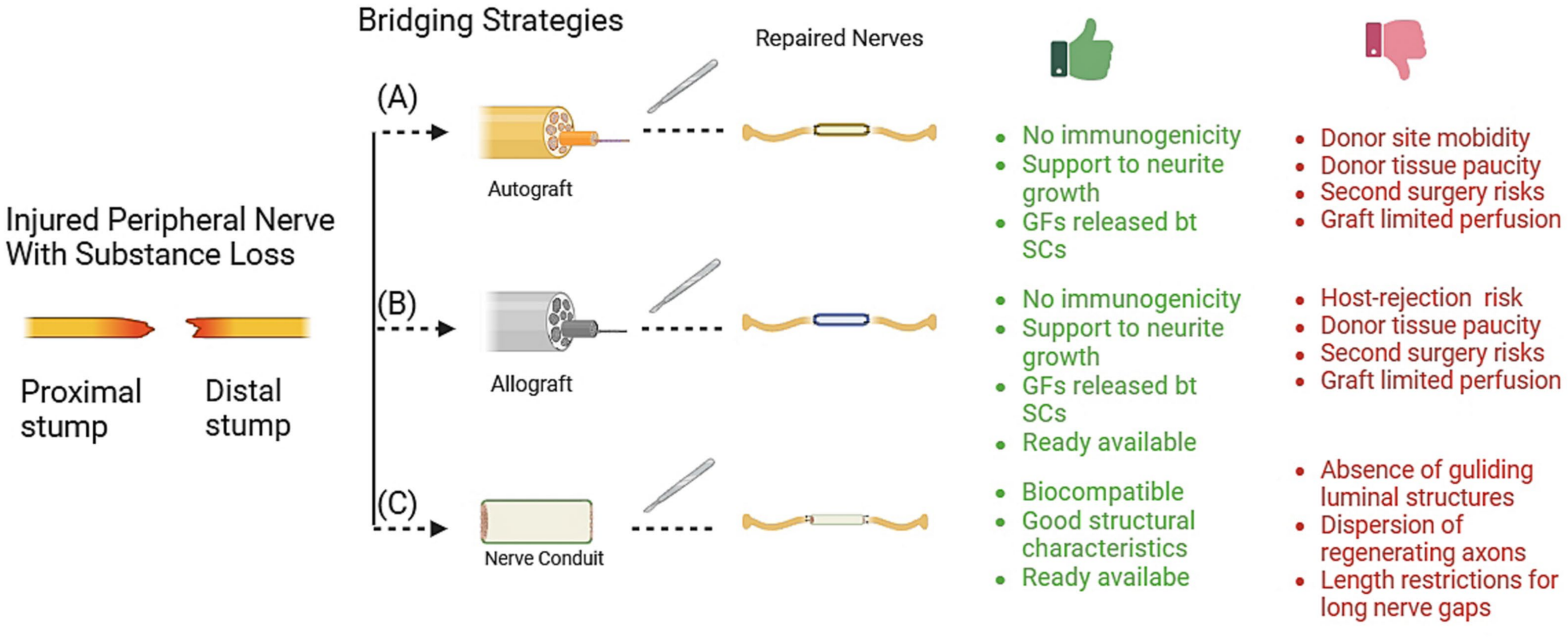
Techniques for bridging peripheral nerve defects. The graphic details the specific advantage (green words) and limitation (red words) for each technique, including (A) autograft, (B) allograft, and (C) nerve conduits. Gf, growth factors; SCs: Schwann cells. Graft materials.

For peripheral nerve gaps that are small in size (<5 mm), the traditional method of suturing repair, without the use of grafted materials, can be employed (14). For longer nerve gaps, different methods of repair have been introduced in the medical field, with varying levels of achievement and acceptance among surgeons. This study aims to provide reference for clinicians in the field of repair techniques and graft materials (see Figure 2).

## Graft materials

### Autologous nerve

Autologous nerve grafts are considered the “gold standard” technique for repair of peripheral nerve defects. Up until now, auttologous nerve transplants have provided the most favorable outcomes in the regeneration of nerves under tension (15). A study reported that autologous nerve grafting provided functional motor recovery in mixed and motor nerve repairs, with meaningful motor recovery observed in 73% of cases (16). However, the availability of autologous nerve grafting is restricted due to limited tissue supply, the requirement for an additional surgical procedure to obtain graft tissue, morbidity at the donor site, loss of function and potential differences in tissue size and structure, etc. (17, 18).

To address the challenges associated with donor site complications, researchers (19) have been seeking an alternative that can match the efficacy of autologous nerves. In a study conducted on SD rats with a 1 cm nerve deficit, the use of vascularized neurotubes for peripheral nerve treatment was investigated. After an eight-week period following the nerve repair procedure, the results revealed that vascularized neurotubes were more effective in promoting nerve regeneration compared to non-vascularized biodegradable conduits and autologous nerve grafts. However, it is important to note that the study had a limited sample size and expanding it will be necessary to enhance the reliability of the research. Additionally, functional recovery was not assessed in this investigation; only histological and electrophysiological markers of nerve regeneration were evaluated. Furthermore, the study did not delve deeply into the vascularization mechanism of the neurotubes, warranting further exploration.

In another study (20), researchers investigated the effectiveness of using minced nerve tissue as a filler within venous grafts to repair 1 cm nerve defects. The study’s findings concluded that incorporating minced nerve tissue into venous grafts significantly enhanced nerve regeneration, comparable to the outcomes of nerve transplantation, without causing complications at the donor site. Consequently, with additional support from experimental evidence and clinical trials, it can be considered a promising alternative for nerve defect repair, potentially replacing the need for autologous nerve grafts.

In peripheral nerve injuries, carefully selecting the most appropriate donor nerve is crucial for successful nerve reconstruction. Several key factors must be considered when choosing a donor nerve, including its function, location, number of branches, and axon count (21). The axon count is particularly important as it helps ensure the transferred nerve can adequately reinnervate the denervated muscle (22). Mackinnon et al. (23) underscored the importance of matching nerves of the appropriate size to optimize the functional outcome following nerve repair, according to their study conducted on an animal model. The research involved nerve transplantation with precise ratios of 1:1, 2:1, and 2.5:1 (donor to recipient axon). In a detailed examination of the forearm, researchers reported [26] that the primary nerve branches of the flexor carpi radialis and the flexor carpi ulnaris had average axon counts of 746 and 659, respectively. These figures were found to align with the average axon counts of the extensor carpi radialis longus (704 axons) and brevis (745 axons). Within this group of wrist flexors and extensors, the extensor carpi ulnaris’ main nerve branch posted the minimum average axon count, at 543. Meanwhile, the primary nerve branches of the supinator, pronator teres, and pronator quadratus presented with average axon counts of 602, 625, and 824, respectively. Axon counts and cross sectional area for lower extremities also were also explored (Table 1).

**Table 1:** Cross sectional area and total axon count of potential nerve donors (24).

Nerve donors	Area, mm^2^	Axons, *n*
Tibialis anterior	0.255 ± 0.111	3,363 ± 1997
Extensor hallucis longus	0.197 ± 0.302	2062 ± 2,314
Flexor hallucis longus	0.234 ± 0.147	1,557 ± 735
Latissimus gastrocnemius	0.256 ± 0.105	2,352 ± 1,249
Medial gastrocnemius	0.309 ± 0.101	2,834 ± 718
Popliteus	0.309 ± 0.101	3,317 ± 1,467
Soleus	0.700 ± 0.222	4,941 ± 1994
Tibialis posterior	0.348 ± 0.253	3,039 ± 1,528
Data presented as mean ± SD		

#### Nerve allograft

In the past, allogenic nerve grafts need to utilize cadaveric or donor nerve tissue as an alternative to autologous nerve grafts (25). However, the disadvantage of using allografts is that it requires systemic immunosuppression lasting up to 18 months (26). In order to avoid the weak, Certain scientists have created modified nerve allografts, which have addressed certain limitations associated with allografts. Certain processing methods, such as multiple freeze–thaw cycles, radiation exposure, and prolonged storage, were utilized to render the allografts non-immunogenic (27). In certain instances, the procedure of identifying a suitable donor, preparing the graft, and subsequently scheduling the surgical intervention could be lengthy (28). Clinical data often suggests immediate surgical intervention yields better results compared to delayed nerve repair (29). Nevertheless, a research study (30) focusing on the application of processed nerve allografts (PNAs) in motor nerve repair unveiled that significant motor recovery was noted in 73% of subjects suffering from mixed and motor nerve injuries in the upper extremities, including the head and neck area. This recovery was observed regardless of whether the repair with processed nerve allografts was performed immediately or in a deferred manner. As an alternative to autologous nerve transplants, companies like Axogen are offering easily accessible frozen decellularized nerve allografts sourced from blood banks. These allografts, post-processing, are preserved at temperatures of either-80°C or 4°C. Recent research (31) suggests that the chosen preservation method could potentially influence motor recovery following nerve reconstruction, with allografts stored in cooler environments kick-starting regeneration earlier than their frozen counterparts. Moreover, the exploration of combining various processing and preservation techniques aims to create an optimal nerve allograft that boasts improved ultrastructural preservation and diminished immunogenicity (32). However, it’s necessary to highlight that acellular allografts necessitate the repopulation of host cells, a process which could potentially result in a delay in axonal regeneration (33). Nevertheless, the regenerative capabilities of extended acellular nerve grafts (processed nerve allografts) are restricted, because SCs do not provide the necessary support for the formation of a basement membrane that contains extracellular matrix (ECM) proteins, which are crucial for axonal growth and the creation of endoneurial tubes that facilitate the growth of regenerating axons (34). However, in clinical applications, decellularized nerve grafts have shown comparable or even superior outcomes compared to other types of transplants. A study conducted on children with obstetrical brachial plexus injury compared acellular processed nerve allograft (ALG) with sural nerve autograft (AUG) and found no significant differences in motor strength and functional components between the two groups (35).

#### Conduits

The rapid development of different materials as a substitute for nerve autografts in mending peripheral nerve injuries has been facilitated by advancements in biomedical techniques. Over the last few decades, research has primarily focused on the use of biomaterial-based nerve conduits for repairing peripheral nerves. These conduits can be made from various materials such as natural substances, non-degradable materials, and biodegradable synthetic material. These conduits possess a longitudinal arrangement that imitates the inherent composition of neural pathways. The conduits serve as pathways for axonal growth, directing regenerated axons to reconnect with their intended neurons. Nevertheless, the channels themselves do not significantly impact the result of neural restoration.

The successful healing of a damaged nerve relies on the gradual processof axonal regrowth and its accurate placement (26). The ideal nerve conduit should possess biocompatibility, biodegradability, flexibility, porosity, pliability, nerve inductivity, and neuroconductivity, with appropriate surface and mechanical properties (36). Most reports on series of nerve conduit reconstructions for digital nerve defects adhereto the boundary of 3 cm. Strauch et al. (37) conducted a study on rabbit sciatic nerve regeneration in which they compared the results of using vein conduits of lengths ranging from 1 to 6 cm. They found that regrowth and functionality were optimal for conduits of lengths ≤3 cm but deteriorated for lengths >3 cm. While nerve conduits are indeed widely used in the repair of certain peripheral nerve injuries, and are often considered effective for nerve gaps smaller than 3 cm, this does not mean that all types of nerve injuries adhere to this rule (38, 39). Firstly, the maximum effective length of a nerve conduit may vary depending on the type of nerve (sensory, motor or mixed) and the specific circumstances of the individual patient (39, 40). Secondly, the material, design, and manufacturing method of nerve conduit might also influence its efficacy in repairing longer nerve gaps (38, 39). Over the years, there have been notable progressions in the development of artificial nerve conduits. A diverse range of novel synthetic polymers and biopolymers have been assessed in terms of materials selection and design.

#### Natural materials

Extensive research has been conducted on the use of organic substances, such as muscle tissue or blood vessels for transporting materials. Natural materials provide greater biocompatibility, lower toxicity, and improved facilitation of cell migration in comparison to synthetic materials (41, 42). A study was conducted to determine whether Schwann cells migrate within nerve conduits used to repair substantial nerve gaps (43). The results revealed that endothelial cells formed a dense network of capillaries, which Schwann cells utilized for migration from both nerve stumps into the conduit. The endothelial and Schwann cells gradually colonized the conduit. A week after the injury, a dense network of newly formed blood vessels was observed encircling both the proximal and distal stumps, with numerous Schwann cells in close proximity. The study concluded that angiogenesis was crucial within the nerve conduits, as it not only aided cell survival but also facilitated the migration of newly developed Schwann cells.

Abundant sources and a lower occurrence of acquired illnesses when collected are additional benefits of utilizing these organic substances. However, in longer nerve defects, the regenerative effects of these conduits on nerves may gradually decrease. In a retrospective case series, Jeon and colleagues assessed 11 patients, all of whom attained acceptable sensory restoration according to both SM2PD and Semmes-Weinstein monofilament examination (44). In their study, Stahl and colleagues examined 28 individuals (28) and discovered that 32% of Siemionow and Sonmez (28) participants attained a sensory improvement of 5–9 millimeters during two-point discrimination testing (45).

Peripheral nerve repair also utilizes non-biodegradable substances like silicone, elastomer hydrogel, or porous stainless steel. The drawbacks of these include inflexibility and instability, potential for causing long-term foreign body reaction and inflammation caused by the formation of scar tissue. These limitations restrict their application in peripheral nerve repair (6). In recent decades, increased attention has been directed towards naturally derived materials utilized in the development of nerve guidance conduits (NGC), peripheral nerve wraps (PNW), and membranes.

These materials should demonstrate biological functionality, sufficient compatibility with living organisms, and the ability to break down naturally (6). Moreover, it is imperative that these substances create structures that closely resemble the extracellular matrix (ECM) and facilitate accelerated tissue regeneration (15).

#### Collagen conduit

Studies have focused on the use of environmentally-friendly substances, including collagen, polyglycolic acid, polylactic acid, polyesters, and chitosan (46). Collagen, a structural protein, is present in the connective tissues of both humans and animals, serving as the main constituent of the extracellular matrix. Implants, such as wound dressings and artificial skin, have made use of it. Natural and biodegradable with low antigenicity, it promotes nerve sprouting, regeneration, and maintains cellular biological functions (17, 38, 47). Among its features are fibers inserted into conduit lumens to function as fillers, as well as hydrogel formulations for the delivery of cells, drugs, and growth factors (17).

A study finding (48) revealed no significant disparities in electrophysiological and hand function outcomes between the collagen conduit and microsurgical neurorrhaphy groups after a 24-month period. Yet, at the 12-month juncture, the collagen conduit group exhibited a statistically significant extension in distal motor latency and a noticeable reduction in compound muscle action potential. A broad-based recovery was observed in both motor and sensory conduction parameters from the 12th to the 24th month. The amplitudes of compound motor action potential regained about 50% of the control hand’s level, the distal motor latency continued to be 50% extended, and a roughly 15% reduction was noted in the motor conduction speed between the elbow and wrist. In conclusion, the data indicates that both collagen conduit and microsurgical neurorrhaphy serve as effective strategies for peripheral nerve repair, delivering comparable outcomes at the 24-month benchmark. In a retrospective case study, Thomsen et al. (49) evaluated 10 patients with collagen conduits for nerves. In the SM2PD test, 50% of patients were classified as “excellent” or “good” in terms of sensation recovery. According to the Semmes-Weinstein monofilament test, at least 80% of patients had recovered light touch sensation. There were no complications reported.

#### Chitosan conduit

The second most abundant natural polymer after cellulose, chitosan is a cationic biopolymer derived from alkaline deacetylation of chitin (50, 51). Recent years have seen extensive use of chitosan in various biomedical fields (51–56), mainly because of its biocompatibility, biodegradability, low toxicity and non-immunogenicity, low cost and large availability. An analysis of chitosan hollow tubes and autologous nerve grafts for reconstruction of peripheral nerve defects was reported by Stenberg L (57). Using chitosan hollow tubes, the authors found that peripheral nerve reconstruction of sciatic nerve in rats was comparable to autologous nerve grafts, the gold standard. According to a study (58), chitosan-based nerve conduits can bridge nerve lesions up to 26 mm in the hand safely and effectively. During early regeneration, tactile gnosis improved significantly, and functional outcomes were similar to those obtained with autologous nerve grafts. Measurement of tactile gnosis using two-point discrimination was the primary outcome parameter. Additionally, a Semmens Weinstein Monofilament Test, self-assessed pain, and a patient satisfaction survey were used as secondary outcome indicators. As a result of complications associated with the chitosan nerve tube, one patient had to undergo revision surgery.

#### Polyglycolic acid conduit

Polyglycolic acid (PGA) is a synthetic polymer that is biodegradable and biocompatible. It has been used in various applications, including orthopedic implants, sutures, and nerve conduits (59). PGA is often used in combination with other materials to enhance its performance. Early studies on synthetic conduits were carried out with PGA. It is recycled, and considered to be more permeable and flexible compared to others, allowing diffusion to help with resorption and regeneration taking place in six months (60). In a prospective level IV case series, Mackinnon and Dellon (61) evaluated 15 patients with digital nerve gaps measuring 17 mm undergoing secondary nerve reconstruction. These researchers discovered that 53% of patients had a good recovery, 14% had a poor recovery, and 33% had outstanding sensory recovery. Sensory nerve grading scales from the British Medical Research Council were used for data collection. In order to qualify for excellent recovery, we required the static two-point discriminability level to be 6 mm, and the moving two-point discriminability level to be 3 mm. These criteria are identical to those used in the commonly used S4 grading system (S0–S4). Movement between 4 and 7 mm and static two-point discrimination between 7 and 15 mm was considered good recovery. The absence of either static or moving two-point discrimination constitutes a terrible outcome. In one case, extrusion was described by Mackinnon and Dellon5, who came to the conclusion that in some sensory lesions less than 3 cm, PGA tubes can produce outcomes comparable to those of the traditional nerve transplant without donor morbidity.

Although several studies mention positive outcomes, some point out that PGA alone has an unfavorable degradation rate for bigger nerve gaps (>3 cm). The method employed to construct the conduit presents another issue. The surface of PGA conduits displayed poor quality when extrusion was applied (62). Additionally, when it breaks down, acidic chemicals are released, causing the pH at the implantation site to drop, which can set off an immunological response. Dehnavi et al. (63) recently reported the findings of a study on the application of a novel neural guidance channel including PGA/collagen/NBG for the enhancement of transected sciatic nerve in a rat animal model. According to the study, the manufactured conduit (bioglass conduit) is more successful in promoting nerve regeneration than PGA and PGA/collagen conduits and has the potential to enhance sciatic nerve regeneration.

The applications of biogradable materials mentioned above have shown similar effectiveness compared to traditional nerve grafts. However, it has been shown that the efficiency of nerve conduits for nerve repair is inferior to that of autograft and allograft when they are employed in digital nerve injury. Results of a systematic review and meta-analysis (64) on methods for repairing digital nerves revealed that all of them produce acceptable results. Nevertheless, autograft and allograft were both superior to conduit repair when treating digital nerve damage with gaps. For static 2-point discrimination (S2PD) outcomes, autograft repair outperformed all other forms of repair statistically, while allograft results generally exceeded neurorrhaphy and conduit repair but were not statistically significant.

Autograft repair statistically outperformed conduit repair and neurorrhaphy for Semmes-Weinstein monofilament testing (SWMF) results while being statistically comparable to allograft repair. Comparing moving 2-point discrimination (M2PD) performance to conduit repair, allograft performed statistically better. Nerve regeneration across large defect gaps has also been demonstrated to be facilitated by nerve conduit lumen fillers (15). As luminal fillers, natural polymers, such as fibrin, collagen, laminin, and agarose, are often used in solutions, hydrogels, filaments, and porous sponges due to their soft properties and biocompatibility. PNI repair and nerve conduit function can be effectively supported by these materials (65). The efficiency of luminal fillers can vary depending on the precise distance of the nerve lesion, despite the fact that many of them have been described. The fundamental criterion for them from the standpoint of clinical translation is that they be conveniently producible and injected into the conduit (65).

#### Conduits with supportive cells

The nerve conduits have recently been improved using a variety of research strategies that speed up nerve regeneration and bridge wide nerve gaps. Supporting cells have been added to nerve conduits, which has attracted the greatest research attention (66, 67). Cell-based therapy is an effective method for mending lengthy nerve defects and can foster the regeneration of peripheral nerves. There are several cell types of interest being studied in this project, including SCs, Olfactory ensheathing cells (OECs), bone marrow-derived mesenchymal stem cells (BMSCs), and adipose-derived mesenchymal stem cells (ADSCs) (68). Augmenting conduits with cells, such as Schwann cells or stem cells, can enhance nerve regeneration by providing cellular support, guiding new nerve fiber growth assisting in myelination, and modulating immune responses (69, 70).

Schwann cells are the most significant and natural seed cells for the healing of peripheral nerve damage. Because they are both structural and functional cells and play a critical role in peripheral nerve regeneration. SCs produce neurotrophic factors such as Nerve growth factor (NGF), brain-derived neurotrophic factor (BDNF), ciliary neurotrophic factor, platelet-derived growth factor, and neuropeptide Y. These neurotrophic components may help injured axons survive longer and encourage their regeneration. It has been demonstrated that transplanting SCs seeds into a nerve conduit can improve axonal regeneration (71, 72). The utilization of autologous stem cells in clinical settings is limited due to various factors, such as the occurrence of morbidity at the donor site, challenges associated with acquiring and rapidly expanding a substantial quantity of stem cells, the requirement for sequential surgical procedures with short intervals (one for harvesting and expanding stem cells and another for nerve gap repair), and the decline in stem cell numbers with advancing age (73). There is a need for some readily available sources of seed cells having SCs properties. The current focus of cell-based therapy research for peripheral nerve injuries is on finding other approaches to SC usage.

It has been demonstrated that astrocytes and SCs share characteristics with olfactory ensheathing cells. Like SCs, OECs produce a variety of neurotrophic substances. In customary conditions, oligodendrocyte precursor cells (OECs) are present in both the peripheral and central nervous systems. Oligodendrocyte precursor cells (OECs) have been employed in clinical trials for the purpose of treating spinal cord lesions in individuals, as well as improving functional recovery in adolescents and young children affected by cerebral palsy. Until recently, their regeneration-promoting function for the peripheral nervous system was unknown (74, 75). The transplantation of olfactory ensheathing cells (OECs) at the time of microsurgical intervention was found to enhance axonal regeneration and improve functional outcomes, as assessed by the sciatic functional index (SFI), in the adult rat sciatic nerve (75). OECs exhibit greater migratory capabilities compared to SCs, and unlike SCs, they do not accumulate proteoglycans, which can result in the collapse of growth cones. As a result, OECs rather than SCs may make a better choice for cell-based regenerative therapy. However, more preclinical and clinical studies are required before OEC transplantation can be used to treat human peripheral nerve injuries (76). Due to their rapid multiplication and ability to integrate into the host in an immunologically safe manner, stem cells are a promising clinically viable alternative to cell-basal therapy for PNI (77). Although embryonic stem cells have the potential to develop into any form of cell, including SC, there are moral questions regarding their usage in medicine. Therefore, researchers have looked for an effective replacement for embryonic stem cells. A more appealing alternative for stem cell therapy is adult stem cells.

A variety of adult tissues can be used to produce mesenchymal stem cells (MSCs), including skin, adipose tissue, bone marrow, and umbilical cord blood. The ability of MSCs to develop into neurons makes them a potential therapeutic target for neurogenesis and neuroprotection. The impact of pre-induced mesenchymal stem cells (MSCs) coated cellulose/collagen nanofibrous nerve conduits on facial nerve regeneration was investigated in a rat model through *in vitro* and *in vivo* experiments, as determined by Cho et al. (78) in their published study.

The findings demonstrated that the regeneration parameters were greatly enhanced by the extra coating of pre-induced MSCs in the cellulose/collagen nanofibrous conduit. According to functional and histological evaluations, Group II, which underwent treatment with the pre-induced MSC-coated cellulose/collagen nanofibrous nerve conduit, exhibited the highest level of recuperation. In each of the three groups, the nerve gap was effectively restored in every rat, and after a period of eight weeks following the surgical procedure, observable degradation of the cellulose/collagen nanofiber commenced. Group II showed a slightly larger nerve diameter than the control group, but there were no neuromas formed, and there was no statistically significant difference in nerve thickness.

Studies have been done on the use of brain-derived neural stem cells (NSCs), in addition to MSCs, in the regeneration of damaged peripheral nerves. An NSC-loaded silicon conduit, which spans the 10-mm gap between the nerve stumps in the study (79), was used to investigate the effects of neural stem cells on sciatic nerve injury in rats. The findings of this study indicate that neural stem cells (NSCs) possess the potential to promote the regeneration of the injured sciatic nerve. Consequently, incorporating NSCs into clinical trials for individuals suffering from nerve injuries could potentially yield improved clinical outcomes, as NSCs have the ability to enhance the expression of nerve growth factor (NGF) and hepatocyte growth factor (HGF) within the sciatic nerve.

Cell-based therapy holds substantial promise, but significant challenges persist in its application in current and future clinical contexts. One such challenge is ensuring the safety of cell transplantation, particularly concerning potential adverse reactions and issues arising in the brain, especially in the case of stem cell transplantation. These issues warrant further investigation. Another obstacle is the extended waiting period required to prepare these autologous cell sources, which could potentially result in missing the critical treatment window (68, 69). SCs and OECs appear to be the most promising due to their inherent roles in nerve function, but limitations include their availability and inconsistent results (80, 81). Mesenchymal stem cells (MSCs) offer a convenient source of cells, but their regenerative capabilities require further exploration (82). The potential of combinatorial approaches utilizing multiple cell types is also under investigation. More comparative research is still necessary to identify the optimal cell therapy approach (see Table 2).

**Table 2:** Summarization of various nerve conduits (39, 83, 84).

Material	Advantages	Disadvantages	Animal trials	Clinical application
Vein grafts	Biocompatible, natural structure	Risk of adhesion/Compression	Dog, primate	Clinical use as grafts
Silicone	Inert, flexible stable	Not biodegradable	Rat, primate	FDA approved
Collagen	Biodegradable, supports regeneration	Potential Immunogenicity, poor strength	Rat, rabbit dog	Limited
Chitosan	Biocompatible antimicrobial	Poor mechanical strength	Rat, rabbit, dog	None
Polyglycolic acid (PGA)	Biodegradable, available in fibers/tubes	Acidic degradation products	Rat, rabbit, dog, monkey	Limited
Nerve conduits with supportive cells	Biocompatible, Supports Regeneration, secrete neurotropic and growth factors	Cell transplant safety, unfavorable reactions	Rat	Limited

A study (85) embarks on an exploration of chemical substances that could potentially foster peripheral nerve regeneration. It seeks to unravel methodologies that could amplify the capacity for nerve repair and regeneration. In addressing this complex issue, the research team meticulously combed through existing literature and experimental data, distilling a selection of chemical agents believed to be conducive to nerve healing, such as neurotrophic and growth factors, and cytokines. The conversation further delves into the realm of cell and tissue engineering therapies, spotlighting the use of nerve scaffolds and conduits, and the innovative application of stem and nerve cells. The culmination of this research is the illumination of clinical applications and future investigative paths, advocating for multidisciplinary and integrated treatment approaches and the exploration of varied therapeutic strategies tailored to different nerve injury scenarios. These research efforts aim to advance the field by enhancing the capacity for nerve regeneration and fostering the progression of nerve repair.

The field of nerve regeneration is witnessing significant advancements in the design and application of implantable biomaterials. Key strategies include the integration of neurotrophic factors, chemical guidance agents, and auxiliary solute factors to foster nerve tissue repair, as well as pioneering bioartificial nerve conduits as novel therapeutic avenues (86). Moreover, the refinement of therapeutic proteins through precise dosage control, optimized release kinetics, and targeted delivery is gaining momentum. Research is also delving into the distinct patterns of angiogenesis and the regeneration across different nerve fiber types. Collectively, these innovations aim to bolster the success rate of nerve regeneration therapies and present enhanced solutions for clinical deployment.

## Technologies to stimulate nerve regeneration

### Electrical stimulation

Electrical stimulation (ES) improves the intrinsic ability of neurons to regenerate in a clinically applicable manner (87, 88). Studies on the peripheral nervous system strongly imply that electrical stimulation has benefits for regenerating sensory and motor neurons (89). In one investigation, DRG cells from chick embryos exposed to an electric field exhibited enhanced neurite development (90). The enhanced growth of peripheral neurons is believed to be attributed to the upregulation of nerve growth-associated genes (such as GAP-43, preprotachykinin A, VEGF, NGF, ANGPT1, CCL11, VEGFC, and Myc proto-oncogene) (91–94), neurotrophic factors such as BDNFs (95), and glial cell line-derived neurotrophic factor (GDNF) (96) in dorsal root ganglia (DRGs). In an randomized controlled trial (RCT) (97), ES demonstrated significant postoperative improvements in all sensory modalities within 5–6 months for patients (*n* = 16) with completely transected digital nerves compared to those who underwent surgery alone (control subjects, *n* = 15). The cold detection threshold for ES patients nearly normalized, achieving 14.33 ± 0.46 just-noticeable difference (JND) units, which was significantly lower than the control group’s 17.22 ± 0.44 JND (*p* < 0.001). Enhancements were also observed in tactile discrimination and pressure detection. Furthmore, the static two-point discrimination in ES patients improved to 4.71 ± 0.90 mm, notably better than the control group’s 8.69 ± 1.05 mm (*p* < 0.001). The duration of electrical stimulation can indeed impact the regenerative capacity of neurons. This is particularly relevant in the context of peripheral nerve injuries, where electrical stimulation has been shown to enhance the intrinsic molecular pathways involved in regeneration, leading to accelerated axonal outgrowth and reinnervation of target tissue (98). However, the timing of electrical stimulation also plays a crucial role. For example, immediate onset of electrical stimulation following surgery has been found to improve functional recovery in cases of large nerve defects in diabetic animals (99). In the field of tissue engineering, electrical stimulation has demonstrated its influence on the behavior of adipose tissue-derived progenitor cells (ATDPCs) in 3D cultures. It promotes the formation of well-connected cellular networks and reduces the diameter of tissue constructs, all while maintaining cell viability and connectivity (100).

In summary, research has demonstrated that electrical stimulation (ES) plays a significant role in promoting axonal regeneration and functional recovery, as well as modulating the biological activity of Schwann cells (SCs), which are essential for nerve regeneration. ES enhances this process by promoting neuronal differentiation, proliferation, neurite outgrowth, and axonal elongation/regeneration, leading to varying degrees of functional recovery in both animals and humans (101). Furthermore, ES influences the behavior of SCs, encouraging their migration, adhesion, elongation, and enhancing their neuronal expression. Interestingly, some studies have found that a direct current of 10 mV is particularly beneficial for the growth and proliferation of SCs (102). It’s important to note, however, that although these studies provide valuable insights, the optimal physical parameters for electrical stimulation, including frequency, intensity, and duration, may vary depending on the specific circumstances and are still subjects of ongoing research (103). Therefore, further studies are needed to establish standardized protocols for the application of electrical stimulation in the context of neuronal regeneration.

### Optogenetic stimulation

In neuroscience engineering, optogenetic stimulation has become a powerful technique. Its great selectivity and lack of invasiveness may exceed the stimulation methods used by its competitors. According to the evidence from various groups, optogenetic activation encourages neurite development (104). Optical pulses and exposure time influence neurite outgrowth and axonal regeneration (104). The study conducted by Park et al. (105) involved the examination of optogenetics, specifically utilizing transgenic Thy1-ChR2-YFP mice expressing ChR228 to generate light-sensitive entire DRGs. The objective of this investigation was to assess the potential enhancement of neurite outgrowth through optically induced neural activity. The researchers explored the impact of various optical stimulation frequencies and exposure durations on neuronal development. In addition, they discovered that the development of optically sensitive neurites was enhanced and skewed in one direction, demonstrating the cell-specific targeting of optogenetics. Indeed, a significant challenge in the application of optogenetic stimulation is its restricted penetration depth, a factor that becomes particularly limiting when addressing peripheral nerve injuries. The ability of light to penetrate tissue is inherently limited, confining the use of optogenetics primarily to superficial structures unless invasive techniques are employed to direct light towards deeper tissues. This constraint becomes especially formidable when trying to stimulate peripheral nerves, which often reside deep within the body. Consequently, while optogenetics presents substantial potential for investigating and treating a range of neurological conditions, its utility in the context of peripheral nerve injuries is presently constrained by the limited depth of light penetration (106).

## Conclusion

For nerve reinnervation, autografts continue to be superior to all bioengineered grafts. However, the drawbacks that result from this point to the requirement for the creation of substitute strategies. In short nerve gaps, the performance of nerve guide conduits made from various materials is comparable to autologous nerve grafts. Most of the bioengineering approaches have been found to focus only on the development of nerve conduits that promote neuronal guidance and growth.

Repairing long nerve gaps remains a significant challenge in the field of nerve regeneration. While there have been advancements in the development of nerve conduits made from various novel materials and the addition of supportive cells, these methods have not yet resulted in a breakthrough for long nerve gap repair.

Additionally, peripheral nerve regeneration techniques using electrical, optogenetic, and magnetic stimulation are showing promise. One possibility is electrical stimulation. However, the standardized parameters for ES have not been established.

While the precise mechanisms underlying the beneficial effects of magnetic stimulation on neurons are not fully understood yet, this technique holds promise as a non-invasive therapy for various neuronal disorders. Furthermore, combining nerve conduits with other peripheral nerve regeneration techniques, such as electrical or magnetic stimulation, could potentially improve the outcomes of long nerve gap repair. However, further research is still needed to optimize these combination approaches, fully elucidate their mechanisms of action, and translate the findings to viable clinical therapies.

## Author contributions

XZ: Writing – original draft. YD: Writing – original draft. AA: Writing – review & editing. HZ: Writing – review & editing. SE: Writing – review & editing. VK: Writing – review & editing. MA: Writing – review & editing. SA: Writing – review & editing. HL: Writing – review & editing. CW: Writing – review & editing.
