# Corrigendum: Techniques and graft materials for repairing peripheral nerve defects

**DOI:** 10.3389/fneur.2025.1591750

**Published:** 2025-06-20

**Authors:** Xiaodi Zou, Yanzhao Dong, Ahmad Alhaskawi, Haiying Zhou, Sohaib Hasan Abdullah Ezzi, Vishnu Goutham Kota, Mohamed Hasan Abdulla Hasan Abdulla, Sahar Ahmed Abdalbary, Hui Lu, Changxin Wang

**Affiliations:** ^1^Department of Orthopedics, The Second Affiliated Hospital of Zhejiang Chinese Medical University, Hangzhou, China; ^2^Department of Orthopedics, The First Affiliated Hospital, Zhejiang University, Hangzhou, China; ^3^Department of Orthopedics, Third Xiangya Hospital, Central South University, Changsha, China; ^4^Zhejiang University School of Medicine, Hangzhou, China; ^5^Department of Orthopedic Physical Therapy, Faculty of Physical Therapy, Nahda University in Beni Suef, Beni Suef, Egypt; ^6^Alibaba-Zhejiang University Joint Research Center of Future Digital Healthcare, Zhejiang University, Hangzhou, China

**Keywords:** peripheral nerve injuries, graft materials, peripheral nerve defects, nerve regeneration, nerve gap

In the published article, author Haiying Zhou was incorrectly affiliated to Faculty of Medicine, The Chinese University of Hong Kong School of Biomedical Science, Shatin, China, it should be Department of Orthopedics, The First Affiliated Hospital, Zhejiang University, Hangzhou, China.

The authors apologize for this error and state that this does not change the scientific conclusions of the article in any way. The original article has been updated.

